# Errors in Tracing Coronavirus SARS-CoV-2 Transmission Using a Maximum Likelihood Tree. Comment on “A Snapshot of SARS-CoV-2 Genome Availability up to April 2020 and its Implications: Data Analysis”

**DOI:** 10.2196/23542

**Published:** 2020-11-11

**Authors:** Peter Forster, Lucy Forster

**Affiliations:** 1 McDonald Institute University of Cambridge Cambridge United Kingdom; 2 Lakeside Healthcare Group at Cedar House Surgery – NHS St Neots United Kingdom

**Keywords:** coronavirus, SARS-CoV-2, genome, phylogenetic, network, tracing, tracking, transmission, dissemination, infection, geography, route

Tracing and quarantining symptomatic and asymptomatic individuals infected by the novel coronavirus SARS-CoV-2 is an important approach to controlling the current epidemic. Tracing the source of an infection can be achieved by conventional interviews, by mobile telephone tracking, or by phylogenetic tracing of the virus genomes themselves, as we have proposed in our work [1].

In a recent critique in *JMIR Public Health and Surveillance* [2] (also see our reply [3]), Mavian and colleagues have disputed our phylogenetic tracing approach and concluded: “it is not possible with the present data to decide which branching pattern (and, therefore, which phylogeographic reconstruction) most likely represents actual dissemination routes among European countries.”

Their underlying reanalysis is, however, based on a trivial oversight. They analyzed genomes collected worldwide in early March 2020 and initially confirmed the B-subclade that we had identified, which links a German sequence to an Italian sequence and thence to further Finnish, Mexican, Swiss, and German sequences. However, they then claim, “in a new tree inferred just one week later, when more than 135 new full genome sequences were made available on GISAID, the direct link between Germany and Italy […] disappeared due to additional clustering of [five] previously unsampled sequences from Portugal, Brazil, Wales, and [two from] the Netherlands.”

Upon request, Dr Mavian provided us with a file of these five new sequences. Comparing these five in our coronavirus sequence alignment table (freely available on the Fluxus Technology website [4]), it transpires that these five sequences are identical to each other and to the pre-existing Italian sequence. Mavian and colleagues [2] appear not to have noticed the identity as they fail to mention it; instead, they present a “maximum likelihood” tree, which misleadingly shows these five new sequences and the pre-existing Italian sequence to be separated by apparently deep branches, even though they are identical. Mavian and colleagues [2] appear to have relied on their computer program without investigating their entered sequences.

Moreover, Mavian and colleagues [2] have not presented the documented patient travel histories of the five new viral sequences. We present these now, using freely available GISAID (Global Initiative on Sharing All Influenza Data) information [5] and contemporary reports, and find that all patients (ie, the Welshman [6,7], both Dutch [8,9], and the Brazilian [GISAID access 412964]) had visited Italy a few days before falling ill. The Portuguese (GISAID access 413648) had visited Spain. Thus, in four of the five new cases, the patient’s travel history to Italy confirms the viral sequence match to the pre-existing Italian sequence. It is therefore unfounded for Mavian and colleagues [2] to claim that the data cannot reveal branching patterns or likely dissemination routes among European countries.

## Abbreviations

GISAID: Global Initiative on Sharing All Influenza Data
